# Granzymes in Neuroinflammation and Neurodegenerative Diseases

**DOI:** 10.1007/s10571-026-01790-y

**Published:** 2026-08-18

**Authors:** Daniele P. Ferrari, Jhunam Sidhu, Karen Jung, Wolfram Tetzlaff, David J. Granville

**Affiliations:** 1 International Collaboration on Repair Discoveries (ICORD) Centre, Vancouver Coastal Health Research Institute, University of British Columbia, 818 W 10th Ave, Vancouver, BC V5Z 1N1 Canada; 2 Department of Pathology and Laboratory Medicine, University of British Columbia, 2211 Wesbrook Mall, Vancouver, BC V6T 2B5 Canada; 3 British Columbia Professional Firefighters’ Burn and Wound Healing Group, Vancouver Coastal Health Research Institute, University of British Columbia, 3891 Main Street Street, Vancouver, BC V5V 3P1 Canada; 4 Department of Zoology, University of British Columbia, 6270 University Boulevard, Vancouver, BC V6T 1Z4 Canada

**Keywords:** Granzymes, Neuroinflammation, Neurodegeneration, Glial cells

## Abstract

Neuroinflammation is a central driver of numerous neurological disorders. While granzymes have classically been studied as cytotoxic effector molecules secreted from immune cells along with the pore-forming molecule, perforin, accumulating evidence suggests that these serine proteases also play roles in non-cytotoxic, neuroinflammatory processes. This review summarizes the current and emerging paradigms related to how granzymes contribute to the progression of neuroinflammation and neurological diseases. Taken together, current research supports a pathogenic role for granzymes as key mediators linking immune activation to neuronal damage and sustained neuroinflammation. Investigating granzyme-modulated neuroinflammatory responses in the CNS may unveil new avenues for therapeutic intervention in neuroinflammatory and neurodegenerative disorders.

## Introduction

### Neuroinflammation

Neuroinflammation is the coordinated immune response of the nervous system, resulting from interactions among neurons, glial cells, as well as resident and infiltrating immune cells. Under physiological conditions, it has a protective role in development, homeostasis, and responses to injury or infection (Mottahedin et al. 2017). However, when dysregulated, neuroinflammation shifts from a reparative process to become a key pathological driver of tissue damage and diseases.

In the central nervous system (CNS), neuroinflammation is primarily orchestrated by microglia, astrocytes and oligodendrocytes. *Microglia*, the resident immune cells, migrate towards sites of injury or infection to engage in phagocytosis and the recruitment of other immune cells. While classically referred to as damage- and disease-associated molecular patterns (DAMPs), it has become apparent that microglia can assume multiple activated states that can be protective or neurotoxic (Paolicelli et al. 2022; Yong 2022; Ma et al. 2024), and are reflected in diverse transcriptomes (Vecchiarelli and Tremblay 2023; Balak et al. 2024). These cells contribute to brain homeostasis and plasticity by releasing neurotrophic factors and clearing cellular debris and pathological protein aggregates, while also engaging in synaptic pruning and tissue surveillance (Gao 2023; Qin et al. 2023; Ransohoff and Cardona 2010). Additionally, microglia respond to a wide range of stimuli and contribute to neuroinflammation through the release of pro-inflammatory cytokines, chemokines, proteases, and reactive oxygen species (ROS) (Escoubas and Molofsky 2024; Yang et al. 2025; Ajoolabady et al. 2025).

*Astrocytes* maintain neural homeostasis by regulating synaptic balance, blood–brain barrier (BBB) integrity, and inflammatory signaling through cytokine and chemokine release, which are dysregulated during neuroinflammation (Lee et al. 2023). Astrocytes show a wide range of diversity reflecting their responses and heterogeneity in CNS pathologies (Escartin et al. 2021; Moulson et al. 2021). Such responses, collectively termed reactive astrogliosis, comprise a heterogeneous set of morphological, metabolic, transcriptional, and functional changes. Besides upregulating inflammatory mediators, reactive astrocytes influence neurons, oligodendrocytes, and other glia through the release of neurotoxic factors, reduced neurotrophic and metabolic support, impaired glutamate uptake leading to excitotoxicity, extracellular matrix remodeling, and contributing to glial scar formation (Patani et al. 2023; Verkhratsky et al. 2023; Ye and Quintana 2025).

*Oligodendrocytes* (OLs) and their precursors (OPCs) are CNS resident cells that contribute to homeostasis; OPCs retain their proliferating capacity and in response to injury, migrate to different brain regions and differentiate into mature OLs. In addition, OPCs directly interact with neurons and endothelial cells, thereby supporting brain and BBB integrity (Fang and Bai 2023). Following differentiation, mature OLs produce myelin and maintain the structural integrity of myelin, which is necessary for neuronal survival. During neuroinflammation, myelin integrity can be profoundly compromised, as proteases released by activated microglia and infiltrating immune cells disrupt OL support and promote myelin degradation (Milward et al. 2007; Spaas et al. 2021; Franklin and Simons 2022). In parallel, oxidative stress and sustained inflammatory signaling impair OPC differentiation required for remyelination, increasing axonal vulnerability and susceptibility to long-term neurodegeneration (Spaas et al. 2021; Franklin and Simons 2022). Moreover, OLs can produce chemokines and pro-inflammatory signals that trigger immune cell activation, perpetuating the inflammatory response (Boccazzi et al. 2022).

In neurological conditions, diverse immune cells such as macrophages, T cells, B cells and mast cells infiltrate the CNS and play critical roles in neuroinflammation. Together, these immune cells form dynamic inflammatory networks that can resolve infection and promote tissue repair or drive chronic neuroinflammatory disease (Ransohoff 2016). Pro-inflammatory cytokines such as tumor necrosis factor-α (TNF-α), interleukin (IL) −1β, and IL-6 are released by activated microglia, astrocytes, and infiltrating immune cells, amplifying inflammation through NF-κB, MAPK, and JAK/STAT signaling pathways, which leads to altered synaptic transmission as well as neuronal injury and dysfunction (Jain et al. 2021; Gadhave et al. 2024). Neurons, in turn, actively shape neuroinflammation by releasing and responding to neuropeptides and inflammatory signals that modulate glial and immune cell responses (Chu et al. 2020).

In neuroinflammatory disorders, the protective responses become dysregulated, shifting from maintaining neural homeostasis to driving pathology. In autoimmune disorders such as multiple sclerosis (MS), aberrant immune activation leads to infiltration of autoreactive T cells and macrophages into the central nervous system, promoting demyelination, axonal injury, and sustained glial activation (Mitchell et al. 2021). Similarly, cerebrovascular conditions such as ischemic stroke trigger robust inflammatory responses in which damaged neurons and vascular cells release danger signals that activate microglia, recruit peripheral immune cells, and disrupt the BBB (Cheng et al. 2023). In neurodegenerative diseases, chronic neuroinflammation contributes to a feed-forward cycle in which sustained immune activation promotes neuronal dysfunction and loss, further amplifying inflammatory signaling (Kempuraj et al. 2020; Giri et al. 2024). Despite the central role of neuroinflammation in disease, current therapeutic strategies remain limited, often targeting downstream inflammatory mediators rather than the upstream mechanisms that initiate and sustain pathological responses (Giri et al. 2024). Among these, proteases have emerged as key regulators of neuroinflammatory processes. Several protease families, including tissue plasminogen activators, matrix metalloproteinases, and neutrophil serine proteases, modulate the extracellular environment through matrix remodeling, regulation of cytokine activity, and activation of cell surface signaling pathways, including protease-activated receptors (PAR) (Almonte and Sweatt 2011; Li and Haney 2020). These functions position proteases as critical mediators of inflammatory amplification and tissue remodeling in the nervous system. Serine proteases, including *granzymes*, represent a particularly important but underappreciated class of regulators in neuroinflammation. Beyond their classical intracellular cytotoxic roles, emerging evidence highlights their extracellular functions in extracellular matrix (ECM) degradation, PAR activation, BBB integrity and cytokine regulation (Mehra et al. 2016; Shlobin et al. 2021). In this review, we discuss recent advances in understanding how granzymes contribute to neuroinflammatory processes in neurological disease and their potential as therapeutic targets.

### Granzymes

Granzymes (*gran*ule-secreted en*zymes*) are a family of serine proteases that contribute to many inflammatory diseases in diverse tissues. The human genome encodes five granzymes (A, B, H, K, and M), each with distinct cleavage preferences and substrate specificity, while mice express ten granzymes (A, B, C, D, E, F, G, K, L, and M) (Richardson et al. 2022). Importantly, substrate cleavage is further defined by the intracellular/extracellular localization for each granzyme, structural conformation of the substrate permitting access to the cleavage site, as well as the presence or absence of protease inhibitors (Huang et al. 2024). Immune cells such as cytotoxic T lymphocytes and natural killer (NK) cells are the common sources for granzymes (Trapani 2001). Other immune (e.g. B cells, dendritic cells, mast cells, macrophages) and non-immune cell types (e.g. keratinocytes, retinal photoreceptor cells, platelets, adipose tissue, type II pneumocytes, chondrocytes) can also express and release granzymes (Richardson et al. 2022). Perforin acts to create a pore through the target cell membrane, facilitating granzyme internalization (Anthony et al. 2010; Souza-Fonseca-Guimaraes et al. 2016). In addition to perforin-dependent internalization, granzymes can also be released and remain active in the extracellular space. We and others have identified granzymes, especially A, B and K, that accumulate extracellularly and may contribute to multiple pathological processes including inflammation, autoantigen generation, endothelial/epithelial barrier dysfunction, bullae formation, impaired tissue remodelling, and ECM cleavage. Consequently, roles for extracellular granzymes have been documented in atherosclerosis, aneurysm, arthritis, autoimmune blistering, dermatitis, delayed wound healing, psoriasis, sepsis, and other inflammatory conditions (Cigalotto and Martinvalet 2024; Aubert et al. 2024; Richardson et al. 2024b), yet their roles in neuroinflammation have not been systematically explored.

#### Granzyme B

Granzyme B (GzmB) is the best characterized of the granzyme family, with a strong cleavage preference for aspartic acid in the P1 position. GzmB/perforin classically induces apoptosis in damaged and infected cells. Dysregulated GzmB activity has been implicated in a wide range of pathological contexts: in autoimmune and inflammatory diseases, including multiple sclerosis, rheumatoid arthritis, and psoriasis, elevated GzmB levels correlate with tissue damage and chronic inflammation (Cigalotto and Martinvalet 2024; Aubert et al. 2024). Consistently, GzmB contributes to pathogenesis through ECM degradation, cleavage of basement membrane and dermal-epidermal junction proteins, and amplification of inflammatory signaling pathways that sustain autoreactive T cell responses and chronic tissue damage (Hiroyasu et al. 2021a; Gleave and Granville 2023; Aubert et al. 2024).

##### Intracellular Roles

GzmB functions as a potent inducer of apoptosis through a perforin-dependent mechanism. Upon target cell recognition, perforin forms transient pores in the target cell membrane, enabling GzmB entry into the cytoplasm. Once internalized, GzmB initiates apoptosis via two coordinated pathways: direct cleavage and activation of executioner caspases, and cleavage of the pro-apoptotic protein Bid into a GzmB-truncated form gtBid. GtBid then induces mitochondrial outer membrane permeabilization and cytochrome c release leading to apoptosome formation, caspase activation, and consequently, apoptosis (Goping et al. 2003; Boivin et al. 2009).

##### Extracellular Roles

Extracellular GzmB exhibits proteolytic activity that is independent of perforin. Multiple substrates have been identified in the extracellular milieu, including ECM proteins (e.g. fibronectin, laminin, vitronectin, and decorin (Cigalotto and Martinvalet 2024), cell-cell junction proteins, hemidesmosomes, proteoglycans, and cytokines (Mouw et al. 2014; Parkinson et al. 2015). Further work supports a role for GzmB in the release of decorin-sequestered transforming growth factor-beta (TGF-β), a cytokine that contributes to microglial homeostasis and neuroinflammation (Li et al. 2024). In addition, GzmB enhances the pro-inflammatory activity of IL-1α and promotes IL-8 secretion. Furthermore, while less understood, GzmB could contribute to the permeabilization of the BBB and/or blood-nerve barrier through the release of Vascular Endothelial Growth Factor (VEGF) and/or cleavage of cell-cell junction and/or basement membrane proteins (Prakash et al. 2014; Matsubara et al. 2020), suggesting a direct role in modulating neurovascular integrity and neuroinflammatory processes.

#### Granzyme K

Granzyme K (GzmK) is a tryptase-like protease that cleaves after basic residues (lysine and arginine). Elevated GzmK levels have been linked to enhanced inflammatory responses and disease severity in several conditions, promoting cytokine production in monocytes and macrophages (Joeckel et al. 2011), and activating the complement system (Donado et al. 2025). Recent studies also identified a distinct subset of GZMK⁺ CD8⁺ T cells that expand during aging and chronic inflammation as well as exhibit low cytotoxicity and strong pro-inflammatory potential of action (Xin et al. 2025), implicating GzmK in systemic inflammation with potential relevance to neuroinflammatory and neurodegenerative processes.

##### Intracellular Roles

Traditionally, GzmK is thought to act intracellularly by inducing caspase-independent cell death. GzmK is released by cytotoxic T cells, NK, and NKT cells, entering target cells through a perforin-dependent mechanism. Once inside, it cleaves intracellular substrates involved in regulating cell death and inhibiting viral replication (Bouwman et al. 2021; de Jong et al. 2021).

##### Extracellular Roles

In the extracellular milieu, GzmK functions as a pro-inflammatory protease (Turner 2025). GzmK proteolytically activates protease‑activated receptors, including PAR‑1 on vascular cells, PAR‑2 on epithelial cells, and PAR-4 in engineered reporter cells, leading to downstream signaling that promotes pro‑inflammatory cytokine release (e.g., IL‑6, IL‑8, and MCP‑1) (Sharma et al. 2016; Kaiserman et al. 2022). GzmK also drives IL-23 secretion by macrophages, which is a critical pro-inflammatory cytokine involved in neuroinflammation and drives proliferation through activation of the MAPK pathway (Richardson et al. 2024a).

#### Granzyme A

Granzyme A (GzmA) is a tryptase, preferentially cleaves at basic residues, and is primarily produced in cytotoxic T cells and natural killer (NK) cells. GzmA stimulates immune cells to produce pro-inflammatory cytokines and is implicated in the pathogenesis of sepsis through the promotion of inflammatory signaling, worsening disease severity (Aubert et al. 2024). Furthermore, in chronic inflammatory diseases, such as Parkinson’s disease and rheumatoid arthritis, GzmA levels are positively correlated with inflammation and disease pathology (Aubert et al. 2024).

##### Intracellular Roles

GzmA contributes to the immune-mediated elimination of target cells, alternatively from classic cytotoxic pathways. GzmA targets a variety of intracellular substrates involved in DNA repair and chromatin organization (Zhu et al. 2006), resulting in cell death through DNA damage, mitochondrial stress, and ROS accumulation. Like GzmK, GzmA has also been reported to induce pro-inflammatory cytokine release, and to induce necrosis when lower levels of perforin are used in experiments (Froelich et al. 2009; Fatima 2025).

##### Extracellular Roles

Extracellular substrates of GzmA include fibronectin, type IV collagen, and proteoglycans, suggesting roles in ECM remodeling and neural tissue integrity (Aubert et al. 2024). GzmA contributes to inflammation by cleaving and activating cytokines such as IL-1β and TNF-α, as well as cell-surface receptors including PAR-1 (Garzón-Tituaña et al. 2021). In the CNS, extracellular GzmA may contribute to autoimmune and infection-associated neuroinflammatory pathology (Arias et al. 2014).

#### Granzyme H and M

In neuroinflammation, granzyme H (GzmH) and granzyme M (GzmM) functions remain poorly characterized. GzmH is a chymase, and GzmM is a metase, with both cleaving after hydrophobic residues. GzmM is involved in context-dependent regulatory roles in inflammation through the promotion of pro-inflammatory cytokines, increased susceptibility to endotoxicosis downstream of TLR4 signaling, and regulation of immune cell recruitment (Anthony et al. 2010; Souza-Fonseca-Guimaraes et al. 2016). GzmH plays a prominent antiviral role, where it cleaves viral proteins and host factors required for viral replication, in addition to inducing fast target-cell death through non-canonical pathways (Andrade et al. 2007; Cigalotto and Martinvalet 2024).

Despite these distinct biochemical activities and roles in inflammation and immunity, there is currently limited evidence linking GzmH and GzmM to inflammatory processes in the CNS. Therefore, this review will focus on the role of GzmB, GzmK, and GzmA in neuroinflammation, starting with the most characterized subtype, GzmB, followed by less described granzymes.

## Experimental Insights for Granzyme-Mediated Neurotoxicity

Accumulating evidence suggests that granzymes induce neuronal death. However, the molecular mechanisms through which these proteases drive neuronal damage and promote neurodegeneration and neuroinflammation remain incompletely understood. Most studies have focused on CD8^+^ T cell-mediated release of granzymes without considering alternative cell sources and the vast number of non-cytotoxic, extracellular mechanisms for granzymes, their potential substrates and downstream consequences that have been elucidated over the past decade. In contrast to GzmB, which has been extensively investigated in CNS injury and neuroinflammation, the roles of GzmA and GzmK remain comparatively understudied, with limited mechanistic and in vivo evidence currently available.

### Granzyme B

**Fig. 1:**
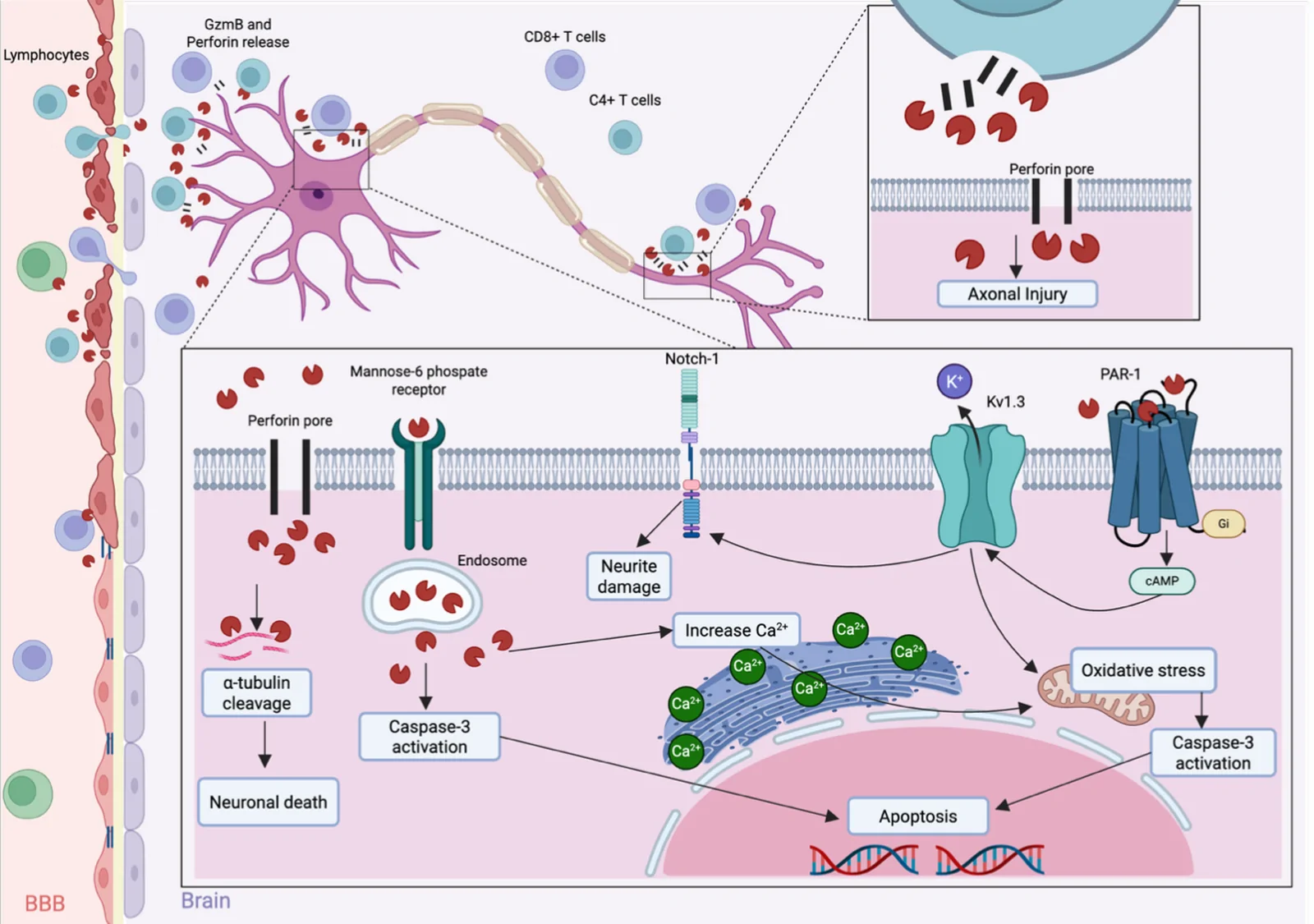
Mechanisms of Granzyme B-mediated neurotoxicity. Activated lymphocytes, including CD8^+^ and CD4^+^ T cells, infiltrating across a compromised blood–brain barrier (BBB) release granzyme B (GzmB) and perforin in the central nervous system (CNS). Perforin-dependent pore formation enables GzmB entry into axons, contributing to acute axonal injury. Within neuronal body, GzmB can enter through perforin pores or via mannose-6-phosphate receptor–mediated endocytosis, followed by endosomal escape into the cytosol. Intracellular GzmB cleaves cytoskeletal substrates such as α-tubulin and activates caspase-3, promoting neuronal death. Extracellular GzmB activates neuronal protease-activated receptor-1 (PAR-1), leading to reduced intracellular cAMP levels, enhanced activation of the voltage-gated potassium channel Kv1.3, and activation of Notch-1 signaling- together driving neuronal dysfunction and apoptosis. These mechanisms converge on calcium dysregulation—characterized by endoplasmic reticulum Ca²⁺ release and mitochondrial Ca²⁺ overload—resulting in DNA fragmentation and neuronal death

A substantial body of literature demonstrates that GzmB is a key mediator of T cell–induced neuronal death across inflammatory and neurodegenerative conditions. In early in vitro studies, GzmB was shown to directly induce neurotoxicity through multiple mechanisms summarized in Fig. 1. However, the pathways by which GzmB enters neurons to exert toxicity are still not completely understood and will be discussed in the section below.

#### Perforin-Dependent and Independent Mechanisms of GzmB Neurotoxicity in vitro

Apart from its classic intracellular role in cytotoxicity, which depends on perforin to enter target cells, GzmB can induce neuronal death through perforin independent mechanisms. Evidence from this comes from studies showing that extracellular GzmB alone is sufficient to induce death of human neurons (Wang et al. 2006; Haile et al. 2011), exhibiting comparable killing activity to that of activated T cells. Consistently, human polarized Th17 lymphocytes expressing GzmB, but not perforin, displayed increased cytolytic activity against human fetal neuronal cells, compared to inactivated T cells (Kebir et al. 2007). These findings establish that perforin is not strictly required for GzmB-mediated neurotoxicity and suggest there may be alternative routes of neuronal entry, which are discussed below.

The role of perforin in neurotoxicity is likely to be dependent on the distinction between neuronal soma or axon injury. Unlike cell body-specific mechanisms, axon-specific injury mechanisms may require direct delivery of active GzmB into the axoplasm by perforin (Sauer et al. 2013). Accordingly, antigen-specific CD8^+^ T cells promoted damage to axons expressing antigen-loaded MHC-I molecules through GzmB activity but did not cause neuronal apoptosis. This occurred independently of caspase signaling, but requires perforin-mediated access to the axoplasm, as axon injury was abolished by GzmB inhibition or perforin deficiency (Sauer et al. 2013). Further insight into the role of perforin can be drawn from studies of antigen-specific CD8⁺ T cell–neuron interactions, using hippocampal neuron co-culture. Meuth et al. (Meuth et al. 2009) demonstrated two temporally distinct effects: rapid perforin-dependent pore formation induces immediate neuronal electrical silencing and Ca²⁺ influx, whereas delayed neuronal apoptosis occurs independently of perforin. Consistently, neuronal death did not differ between wild-type, perforin-deficient, or GzmB-deficient isolated T cells within the early experimental timeframe, indicating that perforin is critical for acute functional impairment but not required for the induction of neuronal apoptosis (Meuth et al. 2009).

Moreover, Haile et al. (Haile et al. 2011) demonstrated in vitro that GzmB can be internalized by neurons via the mannose-6-phosphate receptor (M6PR), accumulating within the neuronal soma, and subsequently escaping from endosomal compartments. Once in the cytosol, GzmB triggers caspase-3 activation and cleaves cytoskeletal proteins such as α-tubulin, culminating in neuronal death. Consistent with this mechanism, blockade of M6PR with mannose-6-phosphate (M6P) prevented GzmB internalization and conferred neuroprotection (Haile et al. 2011). In contrast, another study reported that inhibition of M6PR fails to prevent GzmB-induced neurotoxicity (Wang et al. 2006), suggesting that neuronal uptake via this receptor is not universally required. This discrepancy is hypothesized to be due to dose differences used to inhibit M6PR: the first study used 25mM of M6P and the latter used only 1mM, which may not be sufficient to completely block the receptor. However, although M6PR is likely a tool for GzmB internalization into neurons, previous studies showed that in the absence of perforin, GzmB is not released from endosomes into cytosol (Pinkoski et al. 1998).

In parallel, GzmB released from activated T cells has been shown to induce neuronal apoptosis against differentiated SK-N-SH cells (a human neuroblastoma cell line) through disruption of calcium homeostasis, characterized by cytosolic and mitochondrial Ca²⁺ overload, endoplasmic reticulum Ca²⁺ release, mitochondrial depolarization, caspase-3 activation, and DNA fragmentation (Baratchi et al. 2011). Although this study did not address the role of perforin or the mechanism of GzmB entry, its findings support Ca²⁺ dysregulation as a downstream effector of GzmB-mediated neuronal injury.

Importantly, the neuronal compartment targeted by GzmB-mediated injury is influenced by factors beyond perforin availability. A key upstream determinant is the neuroinflammatory microenvironment, particularly IFN-γ signaling. IFN-γ drives dose-dependent upregulation of MHC-I on axonal membranes through retrograde signaling to the cell body and anterograde transport of MHC-I molecules into distal axons (Sauer et al. 2013; Clarkson et al. 2017). In addition, demyelination can further increase axonal vulnerability by exposing and promoting MHC-I expression in affected regions (Clarkson et al. 2023). Conversely, when MHC-I expression is preferentially localized to neuronal soma, GzmB-mediated cytotoxicity is redirected. This is exemplified in Rasmussen’s encephalitis, where CD8⁺ T cells were found in close apposition to MHC-I–expressing neuronal perikarya and release GzmB in a polarized manner, resulting in neuronal apoptosis (Bien et al. 2002).

#### Extracellular PAR-1 Signaling in GzmB–Induced Neuronal Toxicity In Vitro

An alternative mechanism for GzmB-mediated neurotoxicity is extracellular GzmB signaling through PAR-1. Activation of PAR-1 by GzmB in a culture of human fetal neurons leads to reduced intracellular cAMP levels, increased expression and membrane localization of the voltage-gated potassium channel Kv1.3, and subsequent activation of Notch-1 signaling, ultimately driving neuronal toxicity (Wang et al. 2010, 2012). Notch-1 activation can result in neurite damage as it is associated with tau aggregation and dysfunction and thus may promote degradation of cytoskeletal proteins and consequently lead to neurite atrophy (Lathia et al. 2008). Pharmacological or genetic inhibition of PAR-1, Kv1.3, or Notch-1 in human primary neurons consistently abolished GzmB-induced neuronal damage, establishing this signaling axis as a critical mediator of neurotoxicity (Wang et al. 2012). Notably, Kv1.3 represents an attractive therapeutic target due to its early and pathology-restricted surface expression in neurons (Wang et al. 2020).

Collectively, this evidence supports the concept that GzmB drives neuronal death through perforin-independent mechanisms, involving both extracellular receptor-mediated signaling and intracellular pro-apoptotic pathways.

Apart from T cell-mediated release of GzmB, a decrease in neuronal viability was observed when SH-SY5Y cells were incubated with the supernatant of activated CG-SH cells (a myeloid cell line that secretes perforin and GzmB), while GzmB inhibition attenuated neurotoxicity (Chaitanya et al. 2011) suggesting alternative sources of granzymes in the context of neurodegeneration. Additionally, GzmB impairs not only the survival of differentiated neurons but also decreases proliferation and neurogenesis of neural progenitor cells, indicating broader effects on CNS repair mechanisms. In these cells, extracellular GzmB interacts with a Gi-coupled receptor, inducing downregulation of cAMP–CREB signaling, which is a central intracellular signaling pathway that helps regulate proliferation, differentiation and survival of neuronal progenitor cells (Wang et al. 2010).

#### BBB Permeability

GzmB contributes to CNS pathology by disrupting vascular integrity. GzmB released from activated CD4⁺ T cells compromises BBB integrity by inducing endothelial cell death, thereby facilitating immune cell infiltration and sustaining inflammation (Klotz et al. 2016). Extracellular GzmB further promotes vascular permeability through proteolytic remodeling of the extracellular matrix which results in vascular endothelial growth factor release and amplification of inflammatory responses (Hendel et al. 2014; Jung et al. 2022; Richardson et al. 2022) and through direct cleavage of the endothelial junction protein vascular endothelial (VE)-cadherin, disrupting endothelial barrier integrity (Shen et al. 2016) and likely contributing to BBB dysfunction.

### GzmK

GzmK has recently emerged as a mediator of immune-driven neuronal dysfunction through PAR-1 signaling. GzmK released by CD103⁺ CD8⁺ tissue-resident memory T cells, or purified GzmK induced dose-dependent intracellular Ca2^+^ release in mouse primary hippocampal neurons, and human SH-SY5Y neurons, and this is associated with neuronal alterations (Terrabuio et al. 2025). Excess of Ca^2+^ can cause uncontrolled glutamate release and mitochondrial Ca^2+^ overload, inducing neurotoxicity (Duan et al. 2007). Additionally, GzmK activates PAR-^2^ in sensory neurons, inducing increase in intracellular Ca^2+^ (Hiroyasu et al. 2025). This suggests that GzmK–PAR signaling may represent a mechanism by which immune cells directly impair neuronal function, similarly to GzmB.

### GzmA

GzmA has been implicated in neuroimmune interactions, exerting both acute signaling effects and direct proteolytic activity on neuronal substrates, without a direct effect on neuronal death. An early in vitro study showed that extracellular GzmA induces neurite retraction at nanomolar concentrations through PAR (thrombin receptor) activation, and an antibody-mediated blockade of the receptor abolishes these effects (Suidan et al. 1994).

## Granzyme Modulation of Glial Cells

**Fig. 2:**
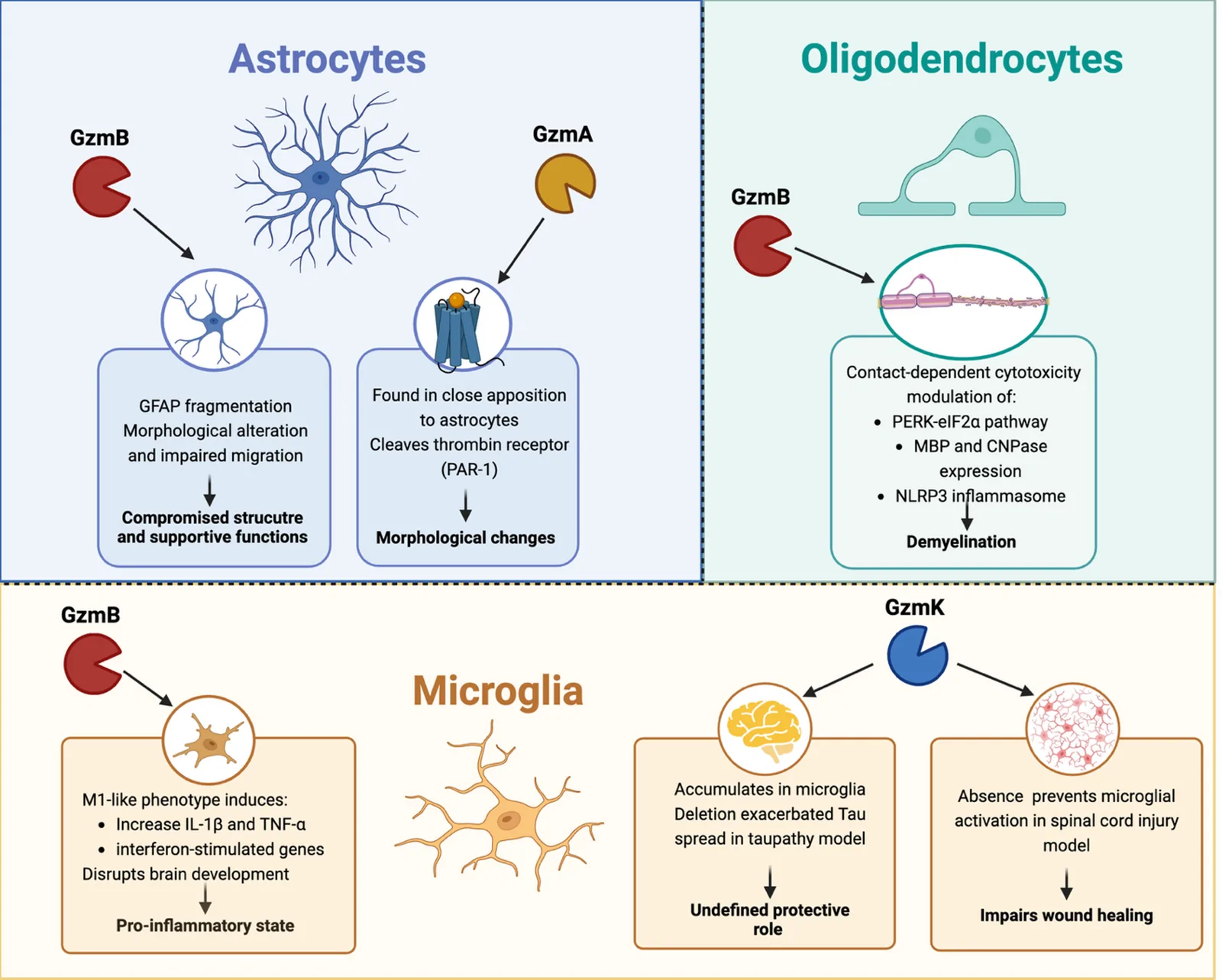
Summary of granzyme interactions with glial cells. Granzymes A, B and K exert distinct effects on major glial populations in the central nervous system. Astrocytes appear relatively resistant to overt granzyme-mediated cytotoxicity, yet are highly susceptible to functional impairment, as T cell–derived GzmB can enter astrocytes, disrupt GFAP integrity, alter morphology, and impair migration without necessarily inducing cell death. GzmA was found in proximity to astrocytes and reversed stellation of these cells. GzmA cleaves thrombin receptor (PAR-1), although its functional effect is still undefined. In oligodendrocytes, GzmB contributes more directly to immune-mediated cytotoxicity and demyelination, particularly through granzyme-dependent T cell subsets and by exacerbating cellular stress pathways such as PERK–eIF2α signaling and inflammasome activation, thereby promoting myelin loss. Beyond direct cytotoxicity, extracellular GzmB acts as a potent immunomodulator of microglia, inducing a pro-inflammatory state and amplifying neuroinflammation in injury and developmental contexts. GzmK accumulates on microglia in neurodegenerative disorders; however its effect is dependent on pathological conditions: the deletion of CD8 ^+^ T cells (major source of GzmK) leads to Tau protein spread, but on the other hand it prevents microglial activation, which improves wound healing and scar formation in spinal cord injury

Glial cells are central regulators of neuroinflammation, as they respond to injury or disease by producing cytokines, chemokines, as well as other inflammatory mediators that shape immune responses and influence neuronal survival and tissue repair (Zhang et al. 2023; Müller et al. 2025). In addition to their direct cytotoxic effects on neurons, granzymes exert immunomodulatory functions by interacting with glial cells (Fig. 2). Comparatively little is known about how granzymes modulate astrocytes, OLs and microglia and the precise mechanisms remain to be fully elucidated. In this section, we compile and discuss the available data showing that granzymes influence glial cells by modulating their activation states and inflammatory signaling pathways during neuroinflammation.

### Granzyme B

#### Astrocytes

Evidence from human disease and experimental models indicates that GzmB can target astrocytes, leading to either cytotoxic or functional consequences depending on the immunological context. For example, in the brain of Rasmussen encephalitis patients (a rare T cell-mediated autoimmune encephalitis), GzmB⁺ T cells were found in close apposition to astrocytes, with polarized GzmB-containing granules oriented toward astrocytic membranes (Bauer et al. 2007), suggesting cytotoxicity. Similar polarization of GzmB at immunological synapses in rat brains has been observed between CD8⁺ T cells and virally infected astrocytes during antiviral immune responses (Barcia et al. 2008). Furthermore, activated CD4⁺ T cells release GzmB in a contact-dependent but perforin-independent manner when interacting with primary astrocytes, leading to GzmB entry into astrocytes and fragmentation of glial fibrillary acidic protein (GFAP), thereby compromising astrocytic structural integrity and supportive functions (Stopnicki et al. 2019). Remarkably, GzmB exposure induced minimal astrocyte toxicity and morphological alterations but instead impaired astrocyte migration in a caspase-dependent manner, effects that were inhibited by pan-caspase inhibition (Wang et al. 2006; Stopnicki et al. 2019). Together, these findings suggest that, while astrocytes are possibly resistant to granzyme-mediated cytotoxicity, they remain highly susceptible to functional impairment by GzmB. This highlights a non-lethal mechanism by which T cells may disrupt glial support and exacerbate neuroinflammation.

#### Oligodendrocytes (OLs)

With regard to OLs, an early in vitro study showed that blocking GzmB activity using γδ T cells did not fully prevent OL lysis within a short incubation period (Zeine et al. 1998). More recently, Th17-polarized human T cells release GzmB upon contact with human OLs in co-culture settings (Jamann et al. 2022). Additionally, extracellular GzmB induced death of primary OLs and MO3.13 oligodendrocyte line, which was prevented by pre-treatment with a reversible GzmB blocker (Ac-IEPD-CHO) and the serine protease inhibitor serpina3n (Jamann et al. 2022). Beyond direct cytotoxicity, mechanistic insights implicate GzmB in OL stress responses and demyelination in a mouse model of diabetes (Yang et al. 2024). In this case, GzmB inhibition by serpina3n prevented demyelination by alleviating endoplasmic reticulum stress through modulation of the PERK–eIF2α adaptive pathway, which further promoted the expression of the myelin-associated proteins: myelin basic protein (MBP) and 2’,3’-cyclic nucleotide 3’-phosphodiesterase (CNPase). Additionally, GzmB inhibition reduced NOD-like receptor pyrin domain-containing protein 3 (NLRP3) inflammasome-mediated IL-1β and IL-18 expression, decreased focal OL death, and ultimately attenuated myelin loss in diabetic mice (Yang et al. 2024). Together, these findings indicate that GzmB contributes to white matter pathology not only by promoting OL injury but also by exacerbating cellular stress pathways that compromise myelin integrity.

#### Microglia

Recent evidence highlights extracellular GzmB as a potent modulator of microglial activation and neurodevelopmental pathology. Transcriptomic profiling in vitro revealed that direct GzmB exposure induces a distinct pro-inflammatory microglial program enriched for IFN-β/IFN-γ responses, antigen presentation, and IL-1β and TNF-α production (Bian et al. 2025), supporting the concept that extracellular GzmB acts as an immune-reprogramming signal rather than solely a cytotoxic effector. Moreover, Bian et al. (Bian et al. 2025) microglial activation mediated by GzmB, leads to neuroimmune disorders in fetuses. In this study, extracellular GzmB released by maternal CD49a⁺ tissue-resident NK cells during maternal immune activation, accumulates in the fetal compartment where it drives interferon-stimulated gene (ISG)–expression in ionized calcium-binding adaptor molecule 1–positive (Iba1^+^) CD68 ^+^ cells, which are markers from macrophage and microglial activation. Genetic ablation of GzmB in maternal NK cells or systemic administration of serpina3n mitigated fetal cortical disorganization, reduced CD68⁺ cell accumulation, and prevented long-term behavioral abnormalities in offspring.

Complementing these findings, an in vitro model of traumatic brain injury showed that damaged adult microglia exhibited increased gene and protein expression of GzmB. This response was reversed by the presence of neonatal microglia, which express high levels of the inhibitor serpina3n (Ye et al. 2025). Serpina3n suppressed GzmB and matrix metalloproteinase (MMP2/9) activity, thereby limiting extracellular matrix remodeling and inflammatory amplification. Additionally, the absence of serpina3n abolished this protective effect, restoring GzmB and MMP activity, reducing levels of IL-10 while elevating IL-1β and TNF-α, thus polarizing microglia toward a pro-inflammatory phenotype. Mechanistically, GzmB can degrade filamin (Browne et al. 2000), a cytoskeletal scaffold required for F-actin polymerization and the structural integrity of tunneling nanotubes (TnTs), which facilitate long-distance intercellular communication and organelle transfer (Keller et al. 2017). Thus, serpina3n-mediated inhibition of GzmB may preserve microglial TnT networks and restrain inflammatory signaling. In conclusion, these studies position extracellular GzmB as a critical driver of microglial activation in both injury and developmental contexts, consistent with our preliminary observations, although the precise receptor-proximal mechanisms by which extracellular GzmB activates microglia remain to be elucidated.

##### Granzyme K

Recent evidence implicates GzmK in tauopathy-associated neuroinflammation through close interactions between clonally expanded CD8⁺ T cells and microglia. During tauopathy progression in humans and mice, CD8⁺ T cells target microglia and deposit GzmK (Mason et al. 2025). Interestingly, depletion of CD8⁺ T cells exacerbated pathological tau spread and accelerated neurological decline, supporting a protective rather than pathogenic role for GzmK in tauopathy. In opposition to the role of GzmB, these findings suggest that GzmK⁺CD8⁺ T cells may function as regulators of microglial homeostasis, limiting the accumulation of dysfunctional microglial subsets and thereby slowing disease progression. However, the precise molecular consequences of GzmK delivery to microglia remain undefined.

GzmK⁺ CD8⁺ T cells were found near Iba1⁺ cells in the injured spinal cord (Kong et al. 2025). In aged mice, these Iba1⁺ cells exhibited increased MHC class I expression—which binds T cell receptor (TCR) and activates CD8⁺ T cells—as well as morphological features of activation, including shorter and fewer branches. In the same study, depletion of CD8⁺ T cells markedly reduced GzmK release and prevented Iba1⁺ cell activation resulting in improved wound healing and reduced fibrotic scar formation in a mouse model of spinal cord dorsal hemisection (Kong et al. 2025).

Collectively, these findings indicate that GzmK⁺ CD8⁺ T cells exert context-dependent effects on microglial function—protective in tauopathy yet potentially pathogenic in spinal cord injury—underscoring the need to define the molecular mechanisms by which GzmK shapes microglial responses across neuroinflammatory conditions. There is no additional literature on the effects of GzmK on other types of glial cells in the context of spinal cord injury.

### Granzyme A

Little is known about the interaction between GzmA and glial cells. In patients with meningoencephalomyelitis, infiltrating CD8⁺ lymphocytes co-expressing perforin, GzmA, and GzmB were found in close proximity to astrocytes within the brain parenchyma. GzmA–positive cells were notably more abundant than GzmB–positive cells, suggesting a prominent role for GzmA-mediated immune responses in GFAP autoimmunity (Guo et al. 2023). Mechanistically, it is known that GzmA activates PAR-1, triggering PAR-1-dependent signaling in astrocytes and neurons—an effect that is blocked by PAR-1-neutralizing antibodies (Suidan et al. 1994; Saito and Bunnett 2005). Together, these findings support a non-canonical role for GzmA as a signaling protease in the CNS, capable of modulating glial and neuronal function independently of direct cytotoxicity.

## Preclinical and Clinical Evidence of Granzymes in Neuroinflammatory Diseases

GzmB, GzmK, and GzmA are frequently elevated in neurological conditions, including neurodegenerative, inflammatory, and traumatic disorders, suggesting a broader involvement of these proteases in neuropathologies. Beyond serving as biomarkers of immune activation, emerging evidence points to the direct involvement of granzymes in neuronal cytotoxicity, disrupting BBB integrity, and modulating inflammatory responses of glial cells, thereby contributing to neurodegeneration and perpetuating inflammatory processes within the CNS.

GzmB is the best characterized granzyme, with particularly strong evidence in support of its role in inflammatory and autoimmune disorders. Elevated levels of GzmB are observed in tissue, serum, cerebrospinal fluid (CSF) and peripheral blood of patients across several neuroinflammatory and neurodegenerative conditions, including MS, Susac syndrome, and amyotrophic lateral sclerosis (ALS) (Malmeström et al. 2008; Ilzecka 2011; Gross et al. 2019a). In addition to GzmB, increasing attention has focused on GzmK as an extracellular pro-inflammatory protease. Nevertheless, its role in neuroinflammatory diseases remains relatively understudied. More recently, increased GzmK expression has been described in the spinal cords of injured patients and in mice with spinal cord injury (Kong et al. 2025). Consistent with this, GzmK levels are elevated in the cerebrospinal fluids of patients with ALS (Kim et al. 2024) and in the brains of individuals with AD (Terrabuio et al. 2025). Similar to GzmK, while GzmA was once viewed as a cytotoxic protease, it likely functions more as a pro-inflammatory mediator, and accumulating evidence indicates that GzmA also plays a role in inflammation-driven immune responses in neurological diseases. A comprehensive summary of granzyme expression in neurological disorders is provided in Table 1.

**Table 1:** The presence of granzymes in neuroinflammatory disorders

Category	Disease	Granzyme	Tissue	Species	Key Findings
Autoimmune inflammatory diseases	Multiple sclerosis	GzmB	Brain; CSF; Peripheral blood	Human	Presence GzmB+ CD8 + in active/chronic active MS brain lesions (Haile et al. 2011; Ifergan et al. 2011; van Nierop et al. 2017); Elevated GzmB levels in the CSF of RRMS (Malmeström et al. 2008) and progressive MS patients (Lee et al. 2017); Increased GzmB+ CD8 + T cells and CD19 + cells in the peripheral blood of RRMS patients (Boldrini et al. 2022); which was positively correlated with disability and progression of MS (Shi et al. 2023); Increased GzmB+ Eomes+ CD4 + T cells in the peripheral blood of SPMS patients (Raveney et al. 2021); Peak of GzmB levels at the conversion period from RRMS to SPMS (Tan et al. 2024).
		GzmK	CSF; Peripheral blood	Human	Increased GzmK+ CD8a + T cells in the CSF of MS patients; GzmK-expressing brain-homing T cells (Koetzier et al. 2022).
		GzmA	CSF	Human	Increased GzmA levels in patients at acute relapse, 1 and 3 months into clinical remission (Malmeström et al. 2008); and around conversion from RRMS and SPMS (Tan et al. 2024).
		GzmH	CSF	Human	Increased levels of GzmH in CSF of MS patients around conversion period to RRMS to SPMS (Tan et al. 2024).
	Autoantibody-associated encephalitides	GzmB	Brain	Human	Increased GzmB+ CD8⁺ T cells in the brain, in close proximity to neurons (Bien et al. 2012).
	Rasmussen’s encephalitis	GzmB	Brain; CSF	Human	GzmB was elevated in the CSF of patients (Takahashi et al. 2009); GzmB+ CD8 + and γδ + T cells cells were found in close apposition to neurons (Bien et al. 2002; Mair et al. 2025).
	Neuromyelitis optica spectrum disorders	GzmB	Peripheral blood	Human	Higher GzmB expression in CD8 + T cells of patients, particularly to the ones with poor response to immunotherapy (Shi et al. 2022).
	Myastenia gravis	GzmB	Thymus	Human	GzmB is aberrantly present in thymus glands from patients and specifically cleaves subunits of acetilcoline receptor (Casciola-Rosen et al. 2008).
Neurodegenerative diseases	Alzheimer’s disease	GzmB	Peripheral blood	Human	Increased GzmB expression in CD4 + T Cells (Kanazawa et al. 2024); CSF levels are reduced (Capelle et al. 2023).
		GzmK	Brain	Human; Mouse	Increased GrK+CD103^–^ CD8 + T cells in the hippocampus of patients and 3xTg-AD mice (Terrabuio et al. 2025).
		GzmA	Brain	Human	GzmA+ CD8 + cells detected in hippocampi from patients (Gate et al. 2020).
	Amyotrophic lateral sclerosis	GzmB	Serum; Peripheral blood	Human	Increased levels of GzmB in the serum of patients, correlated to disease severity (Ilzecka 2011); Increased GzmB + in CD4 + T cells (Kanazawa et al. 2024).
		GzmA	Serum	Human	Elevated GzmA levels (Ilzecka 2011).
	Parkinson’s disease	GzmB	Peripheral blood	Human	Increased GzmB + in CD8 + TEM and TEMRA cells (Capelle et al. 2023).
		GzmK	Peripheral blood	Human	Reduced frequency of GzmK TEM and TEMRA cells (Capelle et al. 2023).
		GzmA	Serum; Brain	Human	Increased GzmA levels in patients in the serum; Increased CD8 + T cells (Galiano-Landeira et al. 2020; Capelle et al. 2023)
	Spinal Cord Injury	GzmK	Spinal cord	Human; Mouse	Increased frequency of GzmK+ CD8 + in spinal cord of injured patients and aging mice (Kong et al. 2025).
	Taupathy	GzmK	Brain; Spinal cord; Lymph nodes	Human; Mouse	Increases GzmK + in phosphorylated Tau lesion in the brain of age-related tauopathy patients; Clonally expanded CD8 + T cells express GZMK in pTau+ brain lesions and spinal cords of P301S+/+ mice (Mason et al. 2025).
Vasculopathies	Susac syndrome	GzmB	Peripheral blood	Human	Elevation of GzmB CD8 + T cells in brain tissue in apposition to endothelial cells; Increased expression of GzmB + and perforin in the blood particularly in the CD8 + TEMRA cell subset (Gross et al. 2019b).
	Ischemic stroke	GzmB	Ischemic brain	Human; Rat	GzmB was elevated in all patient samples and in rat samples, co-localized with dying neurons (Chaitanya et al. 2011).
		GzmK	PBMCs and brain	Human; Mouse	GzmK+ CD8 + T cells increased in peripheral blood of acute ischemic stroke patients, and in the brains of mice after transient middle cerebral artery occlusion (Bai et al. 2025).
Other	Acute encephalopathy	GzmA	CSF	Human	Elevated levels of GzmA in patients compared with complex febrile seizures patients (Yamanaka et al. 2020).

### Autoimmune Inflammatory Diseases

#### Multiple Sclerosis

MS is a chronic, degenerative disease characterized by the infiltration of lymphocytes into the CNS, which provokes the degradation of myelin, inflammation, and consequent neuronal damage. Approximately 80% of patients initially exhibit relapsing–remitting MS (RRMS), the earliest stage of the disease, defined by the appearance of variable neurological symptoms, followed by complete or partial recovery. Within 15 years of the diagnosis, 60% of patients transition to a more advanced stage, secondary-progressive MS (SPMS), exhibiting fewer periods of remission and increasing disability (Scalfari et al. 2014).

##### Granzyme B

In MS, GzmB is strongly associated with disease progression and tissue pathology. GzmB expression within CD8^+^ T cells was detected within MS plaques and white matter lesions, frequently in proximity to OLs and other parenchymal cells (Ifergan et al. 2011; van Nierop et al. 2017). Using RT-PCR, authors observed a 100-fold increase in GzmB transcripts in active MS lesions (defined by demyelination and massive immune cell infiltration) compared with healthy control tissue (Haile et al. 2011). In chronic active lesions (where there is demyelination but decreased lymphocyte infiltration), GzmB levels remain elevated, although this increase is less pronounced than in active lesions, and correlates with CD8⁺ T cell density across lesion types (Haile et al. 2011). Conversely, Fransen et al. (Fransen et al. 2020) did not observe an increase in GzmB⁺ tissue-resident memory T cells in MS white matter lesions, compared to normal appearing areas. However, in this study, GzmB expression was quantified in gated CD69^+^ CD103^+/−^ populations, which highlights potential cell type-specific heterogeneity in GzmB expression.

Increased GzmB protein levels and activity are observed in the CSF of MS patients during clinical relapse, with levels remaining elevated during early remission, despite reduced granzyme expression in circulating peripheral T cells (Malmeström et al. 2008), indicating that cytotoxicity was confined to the CNS rather than reflecting systemic immune activation. Moreover, in patients with progressive MS, GzmB levels in the CSF are nearly 28-fold higher than in healthy controls (Lee et al. 2017). Notably, while levels of GzmB are clearly elevated in CSF and no GzmB inhibitors are known to exist in CSF, little is known about the pathologic mechanism of extracellular GzmB activity in the transition to progressive MS.

In the peripheral blood of RRMS patients, the frequency of GzmB⁺ CD8⁺ T cells is approximately 1.5-fold higher than that of healthy donors (Boldrini et al. 2022), suggesting that cytotoxic function is already engaged early in the disease. Terminally differentiated effector memory CD8⁺ T cells re-expressing CD45RA (TEMRA) are a subtype of T cells that emerged after prolonged or repetitive antigenic stimulation and are widely considered markers of immune senescence and aging (Guo et al. 2023). In this context, TEMRA cells accumulate in SPMS and express high levels of GzmB, which positively correlates with disability scores (Shi et al. 2023). Notably, the abundance of GzmB⁺ TEMRA CD8⁺ T cells discriminates SPMS from RRMS with high accuracy, highlighting their potential value as both biomarkers and pathogenic effectors. Mechanistically, the transcription factor T-bet has been identified as a key regulator driving GzmB expression in TEMRA CD8⁺ T cells in SPMS (Shi et al. 2023). In support, a recent proteomic study identified a distinct GzmB peak in the serum of MS patients that coincided with conversion from RRMS to SPMS. This GzmB elevation coincided with the enrichment of microRNA corresponding to CD4^+^, CD8^+^ T cells and natural killer cells and the depletion of pericyte-associated microRNAs during the conversion to SPMS (Tan et al. 2024). Given the role of pericytes in maintaining the integrity of the blood-brain barrier and limiting immune cell infiltration, these findings suggest a mechanistic link between elevated GzmB, vascular dysfunction, and sustained neuroinflammation.

Importantly, GzmB expression is not restricted to CD8⁺ T cells. CD19⁺ B cells expressing GzmB are 10-fold elevated in the peripheral blood of RRMS patients treated with Natalizumab (Boldrini et al. 2022), pointing to a broader involvement of adaptive immunity in GzmB-mediated pathology beyond classical cytotoxic lymphocytes. Furthermore, an expansion of GzmB^+^ CD4^ +^ T cells in the circulating blood and in the brain has been described in SPMS, accompanied by increased expression of the transcription factor Eomesodermin (Eomes) (Raveney et al. 2021). Eomes has been implicated in the maintenance of chronic neuroinflammation in the experimental autoimmune encephalomyelitis (EAE) model (Raveney et al. 2015). Despite the extensive evidence describing the presence of GzmB in MS, alternative cellular sources and perforin-independent functions of GzmB remain insufficiently explored.

##### In Vivo Mechanisms

Evidence extending from in vitro studies supports a direct neurotoxic role for GzmB in vivo. GzmB detected in the cerebrospinal fluid of patients with progressive MS induces neuronal death through upregulation of neuronal PAR-1 and subsequent mitochondrial dysfunction, an effect potentiated by IL-1β (Lee et al. 2017). In EAE, a mouse model of MS, pharmacological inhibition of GzmB using serpina3n significantly attenuated disease severity and preserved myelin integrity, despite the persistent infiltration of CD4⁺ and CD8⁺ T cells into the CNS in mice post EAE induction (Haile et al. 2015). Although serpina3n also inhibits other serine proteases, these findings support a pathogenic contribution of extracellular GzmB activity.

Beyond direct neurotoxicity, human GzmB may contribute to autoimmune amplification by cleaving transaldolase—an enzyme highly expressed in OLs and decreased at sites of demyelination in MS—thereby generating immunogenic fragments that elicit proliferative and cytotoxic responses of CD8 ^+^ T cell lines from MS patients against OLs (Niland et al. 2010). Together, these studies support a multifaceted role for GzmB in MS pathogenesis, where both intracellular cytotoxic activity and extracellular proteolytic functions may contribute to neuronal damage, OL targeting, and the propagation of chronic neuroinflammation.

##### Granzyme K

In MS, CD8 ^+^ GzmK ^+^ T cells were enriched in the CSF and peripheral blood of patients, and co-expressed the transcription factors RUNX3, Eomes and Tbet, which indicates a brain-homing phenotype (Koetzier et al. 2022). This transcriptional signature is characteristic of cytotoxic effector cells that have migrated across the BBB and persist within inflamed CNS tissue, suggesting a direct contribution of GzmK⁺ cells to neuroinflammation in MS.

##### Other Granzymes

GzmA levels are increased ~ 2-fold in the CSF of MS patients, compared to controls, where levels rise during acute relapse and remain elevated into remission (Malmeström et al. 2008). Similarly, GzmA is elevated in the CSF of patients during conversion from RRMS to SPMS (Tan et al. 2024). Granzyme H is elevated in the CSF during the transition from RRMS to SPMS (Tan et al. 2024). The presence of GzmM in neuroinflammatory diseases has not yet been reported.

### Autoimmune Encephalitides, Rasmussen’s Encephalitis, Neuromyelitis Optica Spectrum Disorder, and Myasthenia Gravis

#### Granzyme B

In autoantibody-associated encephalitides, where the presence of autoantibodies directed against neuronal proteins results in dysregulated adaptive immune responses, the infiltration of GzmB-expressing CD8⁺ cytotoxic T cells is a prominent feature. In these cases, increased appositions of GzmB⁺ T cells to neurons are detected compared to healthy tissues, suggesting active GzmB-cytotoxic effector mechanisms in this condition (Bien et al. 2012). In Rasmussen’s encephalitis—a rare, chronic disorder characterized by persistent unilateral cerebral inflammation and epilepsy—GzmB levels are elevated in the CSF of patients (Takahashi et al. 2009). Moreover, GzmB⁺ T cells, including γδ T cells, are frequently detected in close proximity to MHC class I–expressing neurons, and astrocytes (Bien et al. 2002; Mair et al. 2025; Schwab et al. 2009). Similarly, in temporal lobe epilepsy linked to glutamic acid decarboxylase, GzmB^+^ CD8 ^+^ T cells were detected in close apposition to neurons, with evidence of granule polarization toward the neuronal membrane in cases with both early and prolonged disease (Tröscher et al. 2023), supporting a direct role for granzyme-mediated neuronal toxicity.

In neuromyelitis optica spectrum disorder, characterized by damage to the optic nerves and the spinal cord, GzmB expression in CD8⁺ T cells is significantly increased compared with healthy donors (Shi et al. 2022). In this disorder, high proportions of GzmB⁺ CD8⁺ T cells are associated with poor responses to immunotherapy, suggesting that GzmB may serve as both a pathogenic mediator and a biomarker of treatment resistance.

Myasthenia gravis is a neuromuscular disorder marked by fluctuating skeletal muscle weakness and fatigability caused by impaired neuromuscular transmission. In patients with myasthenia gravis, GzmB is aberrantly expressed in the thymus but not in healthy controls, and GzmB-mediated cleavage of the acetylcholine receptor is hypothesized to initiate or amplify autoimmunity by generating immunodominant epitopes recognized by T cells (Casciola-Rosen et al. 2008). To date, evidence for the presence of GzmA or GzmK in other autoimmune diseases affecting the CNS has not been reported.

### Aging-Related Neurodegenerative Diseases

While increased GzmB and its substrates were listed in the 204 proteins comprising the ‘aging clock’ which was identified by proteomics to predict the risk of mortality and common age-related diseases (Argentieri et al. 2024), and numerous mechanistic roles of GzmB in aging and age-related diseases have been identified (Reviewed in Richardson, Jung, et al. 2022), little is known about the mechanism of action of granzymes in age-related neurodegenerative conditions. Moreover, a distinct clonal population of GzmK^+^CD8^ +^ T cells in humans have been linked to inflammaging, as they correlate with the presence of pro-inflammatory cytokines that promote chronic low-grade inflammation (Mogilenko et al. 2021).

#### Alzheimer’s Disease (AD)

AD is a disorder characterized by cognitive decline and memory impairment, in which chronic neuroinflammation driven by activated glial cells contributes to disease initiation and progression alongside the accumulation of protein as amyloid-β and tau.

##### Granzyme B

Increased frequencies of GzmB⁺ CD4⁺ T cells are detected in the peripheral blood of AD patients (Kanazawa et al. 2024), but in contrast, CSF levels of GzmB are reduced compared to healthy controls (Oláh et al. 2015).

##### Granzyme K

GzmK^+^ CD103^–^ CD8^+^ T cells were increased in vulnerable brain regions in AD, such as the hippocampus, in comparison to the brains of healthy donors (Terrabuio et al. 2025). Similar to human specimens, in 3xTg-AD mice, a transgenic model that mimics the hallmarks of human AD, higher levels of GzmK in the CD103⁻CD8⁺ T cells were reported compared to wild-type mice, suggesting a potential contribution of GzmK⁺ T cells to neuroinflammatory processes in AD models (Terrabuio et al. 2025). The GzmK ^+^ T cells found in AD patients and mice do not express perforin and therefore would suggest an extracellular role of GzmK. Moreover, in chronic traumatic encephalopathy and age-related tauopathy, where there is also an abnormal accumulation of misfolded tau protein, GzmK^+^ CD8^ + ^T cells increased in phosphorylated Tau lesions in the brain of patients and in mice (Mason et al. 2025).

In the context of AD, there is an elevation of PAR-1 expression in hippocampal neurons of 3xTg-AD mice compared to WT animals, establishing the GzmK–PAR-1 axis as a critical immune-mediated pathway linking T cell activity to tau pathology and neurotoxic inflammation in AD (Terrabuio et al. 2025). Additionally, proteomic profiling of differentiated human SH-SY5Y neurons showed that GzmK induces PAR-1–dependent molecular changes associated with neurodegeneration. GzmK upregulated 84 proteins, with enrichment of AD–related pathways, including amyloid fiber formation, MAP kinase–mediated tau hyperphosphorylation, post-translational phosphorylation, and NF-κB signaling, which is associated with ion channel protein expression in neurons, such as Ca^2+^. These effects were largely abolished by pre-treatment with PAR-1 inhibitor SCH79797, that prevented intracellular Ca^2 +^ release in neurons co-cultured with GzmK^+^ CD103^–^ CD8^+^ T cells (Terrabuio et al. 2025). This indicates that GzmK-driven neurodegenerative signaling in neurons is mediated through PAR-1 and highlighting this axis as a potential therapeutic target for AD or possibly other types of neurodegenerative diseases. Additionally, GzmK was recently identified in mice as a memory CD8 ^+^ T cell-derived upstream mediator of aging-related modifications in the brain that leads to hippocampal-dependent cognitive decline. Interestingly, inhibiting GzmK in aging mice improved their performance on a spatial memory behavioral test (Sucharov et al. 2026). Taken together, these findings suggest that GzmK is a promising therapeutic target for age-related neurodegenerative disorders.

##### Granzyme A

In AD, the increase of GzmA⁺ CD8⁺ T cells in the hippocampus of patients, in comparison to control individuals, implicates these cells in localized immune–neural interactions in this disease (Gate et al. 2020). GzmA has been shown to directly cleave tau protein at defined sites, generating N- and C-terminal fragments that become hyperphosphorylated, exhibit aggregation propensity, and seed tau pathology in neighboring cells; notably, tau cleavage products corresponding to GzmA activity are detectable in human tauopathy brains and are fully prevented by pharmacological GzmA inhibition (Quinn et al. 2024). Current evidence supports a model in which GzmA contributes to neurodegeneration through both receptor-mediated signaling and direct proteolytic modification of tau.

#### Amyotrophic Lateral Sclerosis (ALS)

In amyotrophic lateral sclerosis (ALS), a disease that affects upper and lower motor neurons, leading to fatal paralysis, increased GzmB is found in patient peripheral blood (Kanazawa et al. 2024) and in the serum, where GzmB levels correlate with disease severity (Iłz̈ecka 2011). The number of GzmK^+^ effector memory CD8 ^+^ T cells were 15% higher in the CSF of patients with sporadic ALS, compared to the CSF of controls without inflammatory diseases. This subset of T cells was found clonally expanded 7-fold in ALS patients than in controls (Kim et al. 2024). Additionally, GzmA is increased in the serum of patients with ALS, compared to controls (Iłz̈ecka 2011).

In ALS and AD mice, Eomes⁺ Th cells accumulate in the CNS and mediate neurotoxicity through GzmB secretion following recognition of long-interspersed nuclear element-1 (LINE-1) antigen ORF1 presented by MHC class II–expressing microglia (Takahashi et al. 2022). Partial neuroprotection has also been reported in ALS animal models following GzmB inhibition with z-AAD-CMK, although incomplete blockade precludes definitive conclusions regarding efficacy (Coque et al. 2019).

#### Parkinson’s Disease (PD)

Parkinson’s disease (PD) is characterized by the loss of dopaminergic neurons in the substantia nigra and accumulation of α-synuclein pathology.

##### Granzyme B

The frequency of GzmB-expressing cells, along with perforin, is selectively increased in effector memory T cells (TEM) and TEMRA populations within peripheral blood compared with healthy controls (Capelle et al. 2023). Similarly, single-cell RNAseq analysis in CD8^+^ TEM cells showed upregulation of GzmB and perforin in early-to mid-stage PD patients (Capelle et al. 2023), representing valuable peripheral biomarkers for the early diagnosis of PD and possible therapeutic targets. Additionally, in murine models of PD, increased populations of CD8⁺ and CD4⁺ T cells expressing high levels of GzmB and perforin are detected in the spleens of mice and rats (Haque et al. 2020). In rodent models of PD, inhibition of calpain—a calcium-dependent cysteine protease—prevents the upregulation of GzmB in infiltrating CD4⁺ T cells, but not in CD8⁺ T cells. These findings indicate that calpain acts as an upstream regulator of CD4⁺ T cell differentiation and effector function.

##### Granzyme A and K

A reduced frequency of GzmK-expressing TEM and TEMRA cells was found in PD patients’ peripheral blood (Capelle et al. 2023). This could indicate a less exhausted and “younger” phenotype of CD8^+^ T cells in PD patients, considering that elevation of GzmK^+^ CD8^+^ T cells is a hallmark of immune aging (Mogilenko et al. 2021). GzmA is increased in peripheral blood and in CD8⁺ effector T-cell subsets (Capelle et al. 2023), with early disease marked by higher frequencies of GzmA⁺ cytotoxic T cells and later stages characterized by parenchymal CD8⁺ T cells expressing GzmA and GzmK but not GzmB (Galiano-Landeira et al. 2020), which is contrary to previous results.

### Acute CNS Injury

#### Spinal Cord Injury

Spinal cord injury (SCI) is a traumatic or non-traumatic insult to the spinal cord that causes immediate damage to neural tissue followed by secondary pathological processes, including neuroinflammation, glial activation, infiltration of peripheral immune cells, and the release of inflammatory mediators. In the spinal cords of patients with traumatic SCI, an accumulation of CD8⁺ T cells expressing NK cell granule protein 7 (NKG7) and GzmK has been observed in close proximity to the lesion site (Kong et al. 2025). In the same study, increased frequencies of GzmK⁺ CD8⁺ T cells were reported in aged mice under naïve conditions and, more prominently, following SCI, suggesting that GzmK contributes to age-associated neuroinflammatory processes after injury.

In rats, following SCI, GzmB is significantly increased in transected spinal cord regions from day 1 to day 5 post-injury, peaking at day 3 with sustained elevation also detected in the cerebellum compared to sham controls (Chaitanya et al. 2009). GzmB-positive neurons exhibit DNA fragmentation and apoptosis, supporting a direct contribution to cell loss and impaired recovery (Chaitanya et al. 2009).

#### Traumatic Brain Injury

Similarly to SCI, traumatic brain injury (TBI) is a form of acquired brain damage caused by an external mechanical force leading to immediate tissue disruption followed by secondary neuroinflammation. In TBI, effector/memory CD8⁺ T cells expressing GzmB accumulate long-term in mouse brains (Daglas et al. 2019), and in rats, GzmB is upregulated at the trauma site and correlates with CD8⁺ T-cell infiltration (Wu et al. 2021). Increased levels of cleaved GzmB substrates, including caspase-3 activation and PARP provide direct biochemical evidence of active granzyme-mediated proteolysis associated with neuronal apoptosis and neurological impairment after traumatic brain injury in rats (Wu et al. 2021). In this study, 24 h following brain trauma, astrocyte-derived IL-15 further promoted CD8⁺ T cell CNS infiltration and enhanced GzmB expression in rats, revealing a cytokine-driven mechanism that sustains cytotoxic lymphocyte activity (Wu et al. 2021).

Overall, these studies support a pathogenic role for granzymes in traumatic CNS injury, where their sustained expression following SCI and TBI may contribute to neuronal loss, impaired tissue repair, and the maintenance of chronic neuroinflammatory responses.

### Vasculopathies

#### Susac Syndrome

Susac syndrome is a rare autoimmune microangiopathy affecting the brain, retina, and inner ear, in which immune-mediated endothelial injury and associated neuroinflammation drive microvascular occlusion and focal neurological dysfunction. Brain tissue analyses from these patients and from mouse models reveal GzmB-expressing CD8^+^ T cells in direct apposition to MHC class–I-expressing endothelial cells, with polarized cytotoxic granules, strongly indicating that GzmB-mediated cytotoxicity contributes to the characteristic endotheliopathy present in this disease (Gross et al. 2019b). In these patients, GzmB⁺ CD8⁺ T cells were elevated in the peripheral blood compared with healthy donors (Gross et al. 2019b).

#### Ischemic Stroke

Beyond rare autoimmune microangiopathies, GzmB also contributes to neuronal injury in more common vascular insults, including ischemic stroke, which is marked by microglia activation, BBB disruption and immune cell infiltration, exacerbating neuronal damage (Levinson et al. 2025). Indeed, atherosclerotic patients and patients with cerebral ischemia exhibit pronounced elevation in plasma GzmB levels, compared to age and sex-matched healthy controls (Skjelland et al. 2007). Furthermore, within ischemic infarct regions, GzmB is markedly elevated within infarcted brain tissue of patients in addition to the increase of chemokine CXCL10, and co-localized with CD8⁺ T cells penetrating neurons (Chaitanya et al. 2011). Similarly, GzmB also localizes within human neurons in infarct tissue and co-localizes with apoptotic markers such as TUNEL and Annexin V. Mechanistically, GzmB promotes neuronal death through cleavage of anti-apoptotic substrates including HSP70, MCL-1, and PARP-1, a nuclear enzyme involved in cell survival (Chaitanya et al. 2011). Consistent findings were observed in rodent models supporting a conserved role for GzmB in ischemia-induced neuronal damage (Chaitanya et al. 2010).

In acute ischemic stroke, a subset of peripheral GzmK^+^ CD8 ^+^ T cells was identified elevated in patients. These cells infiltrate the brain parenchyma, contributing to neuroinflammation in the early stage of ischemia-reperfusion and correlates with the severity of clinical scores. In transient middle cerebral artery occlusion, a mouse model of stroke, this subset of cells were elevated in the brains, particularly in peri-infarct regions (Bai et al. 2025).

#### Cardiac Arrest/Cardiopulmonary Resuscitation

Within 24 h of cardiac arrest/cardiopulmonary resuscitation (CA/CPR)-induced brain injury in rats, GzmB is elevated and co-localizes with neurons, along with an increase in cleaved caspase-3 and PARP-1. This was reversed by treatment with GzmB inhibitor I, that prevented neuronal apoptosis and cognitive impairment (Ji et al. 2019).

Together, these observations highlight GzmB as a key mediator of vascular and neuronal injury in CNS vasculopathies, linking cytotoxic lymphocyte activity to and neuronal death.

## Translational Challenges and Novel Strategies for Granzyme Inhibition

Genetic inhibition of granzymes by using knockout mice could provide a valuable tool for defining granzyme-specific roles in neurological disease. Studies using GzmA and GzmB knockout mice revealed distinct, non-redundant roles for individual granzymes (Chawla et al. 2024). Similar approaches in neuroinflammatory disease models could help delineate the specific contributions of GzmA, GzmB and GzmK to neuronal injury and neuroinflammation while providing a robust framework for therapeutic target validation in the CNS.

Although serpina3n effectively limits GzmB activity in mice, it remains a suboptimal candidate used for clinical translation: it is only expressed in rodents, raising significant immunogenicity concerns for human use, and it lacks target specificity, inhibiting multiple serine proteases beyond GzmB. In humans, the primary endogenous inhibitor of GzmB is serpinb9 (also known as Proteinase Inhibitor 9, PI-9), a member of the serpin family that neutralizes GzmB intracellularly, thereby preventing unintended cellular damage (Huang et al. 2024). However, serpinb9 is not expressed in neuronal cells and it is restricted to the intracellular compartment and cannot inhibit GzmB once it is released into the extracellular space. Moreover, no endogenous extracellular GzmB inhibitor has been identified in humans to date, underscoring a critical gap in tools for dissecting and therapeutically targeting extracellular GzmB activity.

In contrast, VTI-1002 is a first-in-class small-molecule inhibitor selectively targeting human GzmB, with high potency and minimal activity against caspases and other serine proteases (Shen et al. 2018). In murine models, it improved wound healing and enhanced extracellular matrix organization without adverse effects, supporting its translational potential. More recently, VTI-1002 also reduced disease severity in preclinical models of autoimmune skin disease (Hiroyasu et al. 2021b). However, its translation to neurological diseases could face additional challenges. One consideration is to adequately access the CNS. Although BBB permeability is increased in several neuroinflammatory disorders, the CNS bioavailability of VTI-1002 has not yet been characterized. For this, delivery strategies such as nanoparticle encapsulation may need to be explored. Furthermore, systemic GzmB inhibition could impair physiological cytotoxic immune responses including CD8⁺ T cell- and NK cell-mediated anti-viral and tumor responses, requiring careful evaluation of dosing regimens. Given these properties, VTI-1002 could also serve as a potential pharmaceutical alternative for GzmB inhibition in preclinical models of neuroinflammation, enabling mechanistic studies and therapeutic exploration in CNS disease contexts, although requiring optimization for pharmacokinetics and safety.

Moreover, although pharmacological GzmK inhibitors exist as research tools, they have not yet been tested in CNS disease models. Notably, bikunin, a naturally occurring inhibitor, can inhibit GzmK activity (Wilharm et al. 1999). However, it lacks specificity and can also inhibit other proteases, which limits its utility as a selective research tool. Similarly, endogenous serpins have been identified as inhibitory molecules for GzmA, such as serpinb12 in humans(Niehaus et al. 2015) and serpinb6b in mice (Kaiserman et al. 2014). However, these are also not highly specific and can target multiple serine proteases beyond GzmA. Given the roles of GzmA and GzmK in neuroinflammation and tissue remodeling, there is a clear need to develop novel inhibitors that can selectively target these granzyme subtypes in neurological disorders, although their development will likely face similar challenges related to selectivity, CNS delivery, and preservation of physiological immune functions as VTI-1002.

Differences between human and mouse granzyme repertoires present a significant challenge for the translation of preclinical findings to the clinic. Several granzyme family members differ in their expression patterns, substrate specificities, and biological functions between species, raising the possibility that mechanisms identified in murine models may not fully reflect human biology. Nevertheless, murine genetic models such as single or combinatorial granzyme knockout mice, retain considerable value as experimental tools for studying specific contributions of granzymes to disease pathology in vivo. These complementary perspectives should both be considered when interpreting preclinical research: genetic models offer mechanistic resolution that pharmacological tools cannot yet match, while the translational relevance of findings requires validation in human cells, tissues, and humanized experimental systems. Finally, it is imperative to consider the intracellular versus extracellular mechanisms. While knockout animals offer insights, they lack the capacity to discern between intracellular and extracellular effects of granzymes, which could present different roles in the context of neuroinflammation. For example, intracellular GzmB-mediated apoptosis is advantageous in the context of tumor elimination. However, the accumulation of GzmB extracellularly, resulting from the cleavage of ECM and basement membrane proteins, has the potential to promote metastasis (reviewed by Zhang et al. 2026). Consequently, it would be advantageous to selectively target the extracellular pathway while preserving the integrity of the intracellular pathway. The research about the effect of granzymes in the field of neuroinflammation and neurodegenerative disorders is nascent, and there is still a paucity of studies in this area.

## Implications and Conclusion

Neuroinflammation is now widely recognized as a central driver of neurodegeneration in disorders such as MS, AD, PD, and many others and accumulating evidence positions granzymes as active mediators of this process rather than passive markers of cytotoxicity. Elevated levels of granzymes, particularly GzmB, are consistently observed in neuroinflammatory diseases, where they not only increase within cytotoxic T cells but also accumulate in the extracellular space. Clinically, the enrichment of granzyme-positive T cells in the CSF, peripheral blood or brain tissue aligns with disease severity and progression, particularly in MS. By directly cleaving neuronal and glial substrates or by initiating signaling cascades (e.g., PAR-1/2), intracellular and extracellular granzymes contribute to neuronal and OL death as well as modulation of astrocytes and microglia activation. Targeting granzyme activity — through inhibition of extracellular proteolysis or modulation of cytotoxic T cell responses — may thus represent a promising therapeutic avenue in MS and other neuroinflammatory disorders.

Despite compelling evidence linking elevated GzmB to neurological disease activity and progression, most studies narrowly focus on perforin-associated GzmB expressed by CD8⁺ cytotoxic T cells, implicitly assuming a classical cytolytic mechanism. This focus overlooks several emerging and potentially disease-relevant dimensions of GzmB biology. First, alternative cellular sources of GzmB, including CD4⁺ T cells, B cells, γδ T cells, and possibly innate immune cells, are increasingly reported across neurological disorders but remain poorly characterized mechanistically. Second, perforin-independent actions of extracellular GzmB, such as cleavage of extracellular matrix components, receptors, cytokines, and barrier-associated proteins, are largely unexplored in the CNS, despite strong evidence from non-neurological systems (Reviewed in Aubert et al. 2024; Richardson et al. 2024b). Finally, the temporal dynamics of GzmB expression across disease stages and treatment responses remain insufficiently resolved, limiting its current translational utility as a biomarker or therapeutic target. Addressing these gaps will be essential to fully understand how GzmB contributes to neuroinflammation, neurodegeneration, and tissue remodeling beyond canonical CD8⁺ T cell–mediated cytotoxicity.

GzmK activates PAR-1 expressed in neurons, influencing their function and tau phosphorylation, while also participating in CD8⁺ T cell–microglia interactions that may exert regulatory effects on microglial states during tauopathy. In contrast, GzmA contributes to neurodegenerative pathology through a combination of receptor-mediated signaling and direct proteolytic cleavage of tau, generating aggregation-prone fragments detected in human disease. Together, these findings redefine granzymes as multifunctional immune effectors in the CNS, each with a specific mechanism of action, but that collectively influence neuronal and glial behavior. A deeper understanding of such mechanisms will be essential to determine whether selective modulation of granzyme activity/signaling can be harnessed therapeutically to limit neuroinflammation while preserving beneficial immune functions.

## Data Availability

No datasets were generated or analysed during the current study.
